# The pattern glare and visual memory are disrupted in patients with major depressive disorder

**DOI:** 10.1186/s12888-022-04167-9

**Published:** 2022-08-02

**Authors:** Min Wang, Xiongwei Qi, Xiao Yang, Huanhuan Fan, Yikai Dou, Wanjun Guo, Qiang Wang, Eric Chen, Tao Li, Xiaohong Ma

**Affiliations:** 1 Psychiatric Laboratory and Mental Health Center, West China Hospital of Sichuan University, Chengdu, China; 2 Huaxi Brain Research Center, West China Hospital of Sichuan University, Chengdu, China; 3 Yibin No.4 People’s Hospital, Yibin, China; 4 Department of Psychiatry, The University of Hong Kong, Queen Mary Hospital, Pokfulam, Hong Kong

**Keywords:** Major depressive disorder, Visual memory, Pattern glare

## Abstract

**Background:**

Visual memory impairment is one of the most commonly complained symptoms in patients with major depressive disorder (MDD). Pattern glare is also a distorted visual phenomenon that puzzles patients with MDD. Nevertheless, how these two phenomena interact in MDD remains unknown. This study investigated the association between pattern glare and visual memory in MDD patients.

**Methods:**

Sixty-two patients with MDD and forty-nine age-, sex- and education level-matched healthy controls (HCs) were included in this study. The Pattern Recognition Memory (PRM) test and the Brief Visual Memory Test-Revised (BVMT-R) were applied to measure visual memory. The pattern glare test including three patterns with different spatial frequencies (SFs) was used to explore pattern glare levels.

**Results:**

Patients with MDD scored lower on the PRM-PCi, BVMT-R1, BVMT-R2, BVMT-R3, and BVMT-Rt and higher on the PRM-MCLd than HCs (all *p* < 0.05). Pattern glare scores for MDD patients were higher with mid-SF (*p* < 0.001), high-SF (*p* = 0.006) and mid-high SF differences (*p* = 0.01) than for HCs. A positive correlation between mid-SF and PRM-MCLd scores in all participants was observed (*p* = 0.01, *r* = 0.246). A negative correlation between mid-high difference scores and BVMT-R2 scores (*p* = 0.032, *r* = -0.317) was observed in HCs, but no significant correlation was observed in MDD patients.

**Conclusions:**

The present study showed that visual memory and pattern glare are disrupted in MDD. Visual memory may be associated with pattern glare and needs to be studied in future work.

**Supplementary Information:**

The online version contains supplementary material available at 10.1186/s12888-022-04167-9.

## Background

Major depressive disorder (MDD) is a prevalent and complex syndrome not only characterized by depressed mood, diminished interests, and anhedonia but also accompanied by a wide range of abnormalities in cognition, sensation, and perception [1]. Cognitive impairment is recognized as one of the important symptoms of MDD and is included in the diagnostic criteria [2]. Patients with MDD have consistently demonstrated worse performance than healthy individuals on tests of memory, information processing speed, attention, and executive function [3]. Specifically, memory impairment is one of the most common cognitive impairments in patients with MDD.

Previous clinical studies have established that patients with acute depression show deficits in various memory domains, including but not limited to visual memory, visuospatial working memory, verbal memory, immediate memory, and delayed memory [4–8]. In addition, memory impairment not only affects function during acute episodes of the illness, as recent evidence suggests that cognitive dysfunction persists following symptomatic remission [9]. Recurrent MDD patients displayed residual memory dysfunction even after remission for three years, and persistent memory deficits may be a risk factor for the development of dementia [10]. Moreover, memory deficits during acute and remitted stages of MDD can contribute to symptoms of low mood and reduced occupational and social functioning that have clinical importance [11, 12]. These findings imply that memory dysfunction worsens emotional and social factors associated with MDD, highlighting the need to understand the mechanisms underlying these memory symptoms.

Pattern glare is visual perceptual distortions and / or physical discomforts such as headache, eyestrain, and illusions of color, shape, and motion when viewing repetitive striped patterns [13]. Pattern glare is usually assessed by square-wave gratings with even width and spacing, and high contrast [13]. Abnormal pattern glare has been revealed to be associated with migraine [14], stroke [15], and autism [16]. Some visual perception abnormalities, such as diminished perception of ambient light [17], photophobia [18], dysfunction of pre-attentive visual information processing [19], deficits in visual surround motion suppression [20], and reduced visual contrast suppression [21], have been observed by an abundance of studies in MDD patients. Our previous study indicates that MDD patients have elevated pattern glare [22].

Pattern glare shares some neural mechanisms with visual memory impairment. Pattern-provoked visual distortions and discomfort have been found to be associated with visual cortical hyperactivation caused by a stressful striped pattern [14, 23]. Further evidence indicates that visual cortical functional connectivity is indicated to be related to visually stressful striped patterns [24]. In addition, both pattern glare and visual memory are regulated by γ-aminobutyric acid (GABA). It has been known that lower GABA is associated with impaired visual memory, while the effect of GABA on pattern glare is greater under binocular conditions than under monocular conditions [13, 25].

MDD patients have abnormal activation of the visual-related cortex. A previous functional neuroimaging study of the brain suggested alterations in activity in visual association areas, including the occipital lobe, lingual gyrus, and fusiform gyrus, in those with MDD [26, 27]. Emerging evidence has indicated altered brain asymmetry in depressed individuals; thus, the lateralized and efficient visual processing system is disrupted when processing visual information [28]. On the one hand, it has been revealed that short-term visual memory depends on coding and activity in the visual cortex [29]. Impaired visual working memory in individuals with MDD has been found to be associated with alterations in the prefrontal cortex and amygdala and in visual cortex activation [26]. Reductions in hippocampal volume have been the most replicated findings and demonstrate that the hippocampus is associated with visual memory impairment in individuals with MDD [27, 29]. On the other hand, MDD is caused by a weakening of excitatory synapses in multiple brain regions [30]. Preclinical studies have indicated that adult GABA-dependent neurogenesis in the hippocampus is correlated with memory acquisition [31]. Reductions in inhibitory GABA levels have been observed in vision-related cortices in patients with MDD [32, 33]. Song et al. demonstrated that reductions in occipital GABA were related to impaired visual perception in acute depressive episodes [20].

Furthermore, colored tints or filters have been reported to reduce perceptual distortions, headaches, and discomfort from striped patterns and improve reading in dyslexia [14, 34]. Nevertheless, it is unclear whether visual memory is related to pattern glare.

Therefore, we hypothesized that MDD patients have abnormal visual memory and pattern glare scores and that visual memory is associated with pattern glare scores in MDD. To test this hypothesis, we analyzed the differences in visual memory and pattern glare levels between patients having MDD and healthy controls (HCs). We also analyzed the correlation between pattern glare and visual memory in attempt to identify the underlying mechanism of visual memory impairment.

## Methods

### Participants

The study recruited 62 inpatients with MDD (38 women, mean age 25.77 years) from the Mental Health Centre of West China Hospital, Sichuan University, between September, 2019 and December, 2020. All patients were Han Chinese between the ages of 18 and 60 years. The MDD participants were diagnosed based on the Diagnostic and Statistical Manual of Mental Disorders, fifth edition (DSM-V) and met the criteria for a depressive episode in the Structured Clinical Interview for DSM-V (SCID). MDD patients had never received anti-depression treatment or stopped treatment for at least 3 months or have taken drugs for no more than 3 days at the time of recruitment. Patients were excluded if they 1)　had any serious physical diseases, especially nervous system diseases; 2) had a comorbid axis I disorder; 3) could not complete the test because of problems such as eye disease, impaired vision, or color acuity; 4) had received electroconvulsive therapy; or 5) were pregnant or lactating. The 17-item Hamilton Depression Rating Scale (HAMD) was used to measure the severity of depressive symptoms.

Forty-nine age-, sex- and education level-matched HCs (32 women, mean age: 26.45 years) who had no axis I mental disorder according to SCID-NP were recruited. Other inclusion and exclusion criteria were consistent with the MDD group.

All participants were informed about the details of the study, and written consent was obtained. The Ethics Committee of West China Hospital of Sichuan University approved this study.

### Visual memory tests

Visual memory was assessed with the Pattern Recognition Memory (PRM) [35] test from the Cambridge Neuropsychological Test Automated Battery (CANTAB) assessment and the Brief Visual Memory Test-Revised (BVMT-R) from the Wechsler Adult Intelligence Scale-revised China (WAIS-RC) [36].

The PRM task consisted of two phases. At first, the participants were presented 12 colored shapes, one at a time, followed by pairs of shapes, one new and the other previously viewed. The participants had to select the previously viewed shape from each pair. After that, another 12 new shapes were presented in turn, and the participants were required to identify them after 20 min. Outcome data included percentage of correct responses in the immediate (PRM-PCi) and delayed (PRM-PCd) tests and the mean correct latency to responses in the immediate (MCLi) and delayed (MCLd) tests.

The BVMT-R is a measure of visuospatial short-term memory and learning. The participants needed to memorize six geometrical figures and their precise shapes and locations during the 10-s presentation. In addition, they needed to draw the memorized figures in the right location immediately afterwards, but the time they spent on drawing was not limited. Based on the accuracy of the shape (1 point) and location (1 point) of the figure, a score from 0 to 2 points for each figure was recorded. The procedure was repeated three times, and the total recall score consisted of the sum of the individual scores from the three trials. Outcome measures included the scores of the three tests and the total score: BVMT-R1, BVMT-R2, BVMT-R3, and BVMT-Rt.

With the exception of MCLi and MCLd, higher scores of the indicators indicated better visual memory performance.

### Pattern glare test

Before the pattern glare test, the individuals were tested for visual acuity and color vision with the Snellen visual acuity chart. Individuals with abnormal visual acuity and color vision were excluded from this study. The present study employed the same pattern glare test as our previous investigation [22].

There were three types of pattern glare stimulation that differed in terms of their spatial frequency (SF). The three separate SFs in order of viewing included a low-SF pattern of 0.3 cycles per degree (cpd), a mid-SF pattern of 2.3 cpd and a high-SF pattern of 9.4 cpd. The low-SF pattern served as a control and at the same time ensured that the participants provided accurate responses. The mid-SF pattern was the main test, which was designed to elicit maximum visual discomfort. The high-SF pattern served as another control and was expected to generate fewer distortions than the mid-SF pattern. Viewing distance was approximately 40 cm[15]. Ambient light was sufficient to allow participants to view the printed glare tests clearly. For every presentation, the participants were allowed to concentrate on a small fixation dot in the middle of the picture for 5 s. They were asked to report which of the following 15 experiences were perceived: colors, including red, green, blue and yellow; bending, blurring or flickering of lines; shadowy shapes among the lines; fading; pain; nausea; dizziness; unease; discomforts; and other. The number of these perceived experiences reported was summed to provide a pattern glare score of each pattern [37]. The mid-high SF difference variable was obtained by subtracting the mid-SF score from the high-SF score and was also included in the analysis [38]. Participants with higher pattern glare scores were supposed to have more severe pattern glare [13].

### Statistical analysis

Group comparisons for demographic, visual memory, and pattern glare were examined between MDD patients and HCs. The independent samples t-test was used for continuous variables (age, education year, visual memory, and pattern glare), and the χ^2^ test of independence for categorical variables (sex). Data were expressed as the mean and standard deviation (SD) for continuous variables. Partial correlation analysis was utilized to analyze the correlations between visual memory and pattern glare separately in MDD patients and HCs, and all participants; and age, sex, and education year were used as covariates. The false discovery rate (FDR) was controlled in multiple tests, and adjusted p value that less than 0.05 was deemed to be statistically significant [39]. Effect size (Cohen *d*) was reported for continuous variables. Statistical analyses were performed using SPSS 26.0 (IBM, Armonk, NY, USA).

## Results

### Demographic characteristics

No significant differences were observed between the MDD patients and HCs in terms of age, gender or education years (all *p* > 0.05). The HAMD score of MDD patients was 20.24 ± 3.778 (Table 1).

**Table 1:** Differences in demographic characteristics between the MDD patients and HCs

Items	MDD (*N* = 62)	HCs (*N* = 49)	t/χ^2^	p
**Age (years)** ^a^	25.77 ± 7.715	26.45 ± 7.792	0.456	0.650
**Sex (male/female)** ^b^	24/38	17/32	0.189	0.696
**Education year (years)** ^a^	14.42 ± 2.385	15.39 ± 3.061	1.874	0.064
**HAMD total score**	20.24 ± 3.788	-	-	-
**First episode (Y/N)**	27/36	-	-	-
**Episode times**	2.90 ± 2.890	-	-	-
**Total duration (months)**	51.10 ± 67.97	-	-	-

^a^ two-sample t-test

^b^ χ^2^ test

Data presented as mean ± standard deviation

*Abbreviations*: MDD, major depressive disorder; HCs, healthy controls; HAMD, Hamilton Depression Rating Scale

### Differences in visual memory between MDD patients and HCs

MDD patients had significantly lower PRM-PCi (t = 7.535, Cohen’s *d* = 0.601, *p* = 0.014), BVMT-R1 (t = 5.703, Cohen’s *d* = 0.476**,** p = 0.025), BVMT-R2 (t = 7.512, Cohen’s *d* = 0.532, *p* = 0.014), BVMT-R3 (t = 7.647, Cohen’s *d* = 0.570, *p* = 0.014), and BVMT-Rt (t = 10.079, Cohen’s *d* =  = 0.632, *p* = 0.014) scores and higher PRM-MCLd scores (t = -6.058, Cohen’s *d* = 0.482, *p* = 0.024) than HCs. All p values were corrected by FDR. No significant differences were found in PRM-MCLi and PRM-PCd scores between MDD patients and HCs (Table 2).

**Table 2:** Comparison on visual memory between MDD patients and HCs

Items	MDD (*N* = 62) Mean (SD)	HCs (*N* = 49) Mean (SD)	t	Cohen’s *d*	p^*^
**PRM-PCi**	87.37 (12.42)	93.71 (7.52)	7.535	0.601	**0.014**
**PRM-MCLi**	3256.10 (1715.38)	2685.69 (1612.64)	-1.874	0.341	0.199
**PRM-PCd**	71.39 (12.59)	75.17 (14.97)	1.365	0.276	0.245
**PRM-MCLd**	2609.06 (991.40)	2216.40 (506.17)	-6.058	0.482	**0.024**
**BVMT-R1**	2.05 (2.18)	3.22 (2.78)	5.703	0.476	**0.025**
**BVMT-R2**	5.24 (2.65)	6.73 (2.98)	7.512	0.532	**0.014**
**BVMT-R3**	7.56 (2.92)	9.16 (2.66)	7.647	0.570	**0.014**
**BVMT-Rt**	14.85 (6.56)	19.12 (7.09)	10.079	0.632	**0.014**

p^*^ corrected by FDR correction

*Abbreviations: MDD* Major depressive disorder, *HCs* Healthy controls, *PRM* Pattern Recognition Memory, *BVMT* Brief Visual Memory Test-Revised

### Differences in pattern glare between MDD patients and HCs

**Fig. 1:**
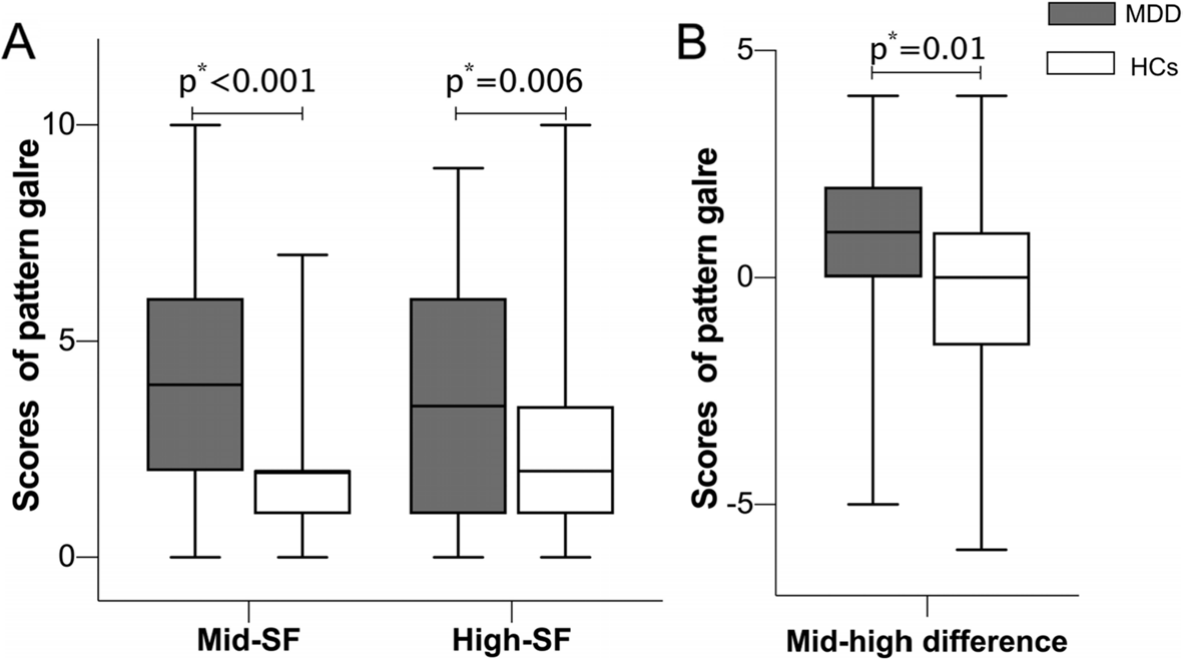
Comparisons of pattern glare scores between MDD patients and HCs. p*, corrected by FDR correction. Abbreviations: MDD, major depressive disorder; HCs, healthy controls; BVMT, Brief Visual Memory Test-Revised

MDD patients had higher scores than HCs when viewing the mid-SF grating (t = 6.01, Cohen’s *d* = 1.15, *p* < 0.001) and high-SF grating (t = 2.97, Cohen’s *d* = 0.56, *p* = 0.006) and with the mid-high difference scores (t = 2.70, Cohen’s *d* = 0.52, *p* = 0.010). All p values were corrected by FDR (sTable 1, Fig. 1).

### Correlations between pattern glare and visual memory

**Fig. 2:**
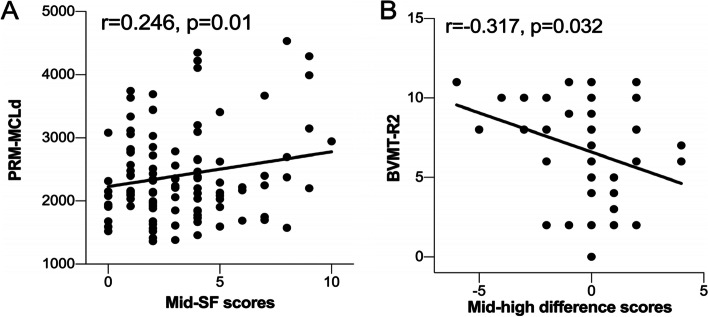
Correlations between pattern glare and visual memory. **A** Correlation between mid-SF and PRM-MCLd scores in all participants. **B** Correlation between mid-high SF differences and BVMT-R2 scores in healthy controls

In the present study, mid-high difference scores were negatively correlated with BVMT-R2 scores (*p* = 0.032, *r* = -0.317) in HCs after controlling for age, sex, and years of education; but no association was observed in MDD patients. In addition, Mid-SF scores were positively correlated with PRM-MCLd scores (*p* = 0.010, *r* = 0.246) in all participants ( sTable 2, Fig. 2).

## Discussion

In this case–control study involving 62 MDD patients and 49 well-matched HCs, we analyzed the difference in pattern glare and visual memory between MDD patients and HCs. Our results suggested that MDD patients have impaired visual memory and elevated pattern glare, and elevated pattern glare is associated with visual memory impairment in HCs.

### Visual memory impairment in MDD patients

In agreement with previous studies, MDD patients in the present study demonstrated deficits in visual memory [7, 40, 41]. Consistent with our previous work, impairments in delayed visual spatial memory were identified in unmedicated MDD patients [42]. Both visuospatial learning and memory were impaired in young adult outpatients with MDD [43]. MDD patients without anxiety have better cognitive function than those with anxiety measured by the BVMT-R [36]. A previous study confirmed significant improvements in visual memory after treatment [40]. In line with an article demonstrating that processing speed in MDD patients is slower than that in HCs, we also found significant differences in the PRM-MCLd (correct latency to responses) between MDD patients and HCs [44].

Studies on the structure and function of the brain have revealed some possible explanations for the relationship between visual memory impairments and depression. An accumulating body of studies has reported that MDD patients have volumetric alterations in the hippocampus that are closely related to visual memory impairments [8, 45]. Decreased levels of GABA in the occipital lobe were related to impaired visual perception in acute depressive episodes [20]. The lateralized and efficient visual processing system was disrupted when processing visual information by deficits in brain asymmetry [28].

### Elevated pattern glare in MDD patients

Our results showed that MDD patients have an elevated pattern glare. In agreement with our previous work, the patients with MDD scored higher in the mid and high SFs than HCs [22]. Similar findings were reported by Golomb et al., who applied a perceptual task designed to be similar to pattern glare models. They found that MDD patients had enhanced motor perception for typical inhibitory stimuli compared with the HCs, indicating that MDD patients exhibited decreased spatial suppression [46]. This result was replicated by Song et al. who demonstrated deficits in visual surround motion suppression in acute MDD patients [20]. Various findings have shown that the balance of excitation and inhibition mediates visual spatial suppression [47, 48]. Spatial inhibition occurs when the contrast of visual information is high, which reflects the antagonism of central and peripheral nerves in the middle temporal visual area [49].

The reduction of GABA in the visual cortex is thought to result in an activated visual cortex [46]. GABA reductions were found in the occipital cortex of untreated MDD patients in remission, the anterior cingulate cortex of young MDD patients, and the anterior lateral / medial prefrontal lobe of MDD patients [50–52]. Decreased GABA in the visual cortex of individuals with MDD may lead to an enhancement of visual cortex excitability.

### Relationship of pattern glare and visual memory

The present study indicated that pattern glare has a negative effect on short-term visual memory in healthy individuals. Pattern glare is visual perceptual distortions and / or physical discomforts. One with elevated pattern glare will experience visual perceptual or physical discomfort when viewing repetitive stripes [13]. Distractions when representing information can damage storage mediated by the visual cortex and lead to disruptions in working memory [53]. Therefore, individuals with elevated pattern glare may have an accompanying visual memory impairment in conditions where the brain’s essential function is coordinated and stable.

Decreased GABA and hyperexcitability in the visual cortex seem to partly explain the relationship between elevated pattern glare and impaired visual memory in those with MDD [25]. Nevertheless, no significant association was observed between visual memory and pattern glare in MDD patients in the present study. Such results may be due to the limited sample size and heterogeneity of MDD.

Symptoms associated with a high level of pattern glare can be relieved by color filters or colored glasses [14]. We found that high-level pattern glare may be associated with visual memory impairment in HCs. Colored covers can be considered in some special conditions, such as the need for improved memory of visual information containing repetitive stripes [54]. Further research is needed to verify this recommendation for depression.

Despite the findings aforementioned, the present study has several limitations. First, we had a relatively small sample size, which in part resulted in no significant relationship between pattern glare and visual memory in MDD patients. Second, also because of the small sample size, we were unable to further divide our participants into subgroups for more specified analysis. In a sense, however, we have tried to minimize the medication impact on both visual memory and pattern glare by choosing currently unmedicated MDD patients although they had been on medication before. In the future, we may conduct similar studies on the first episode, drug-naive MDD patients and those having recurrent depressive disorders to minimize bias. Third, we did not explore the exact mechanism of GABA and neuroimaging. Future researchers may take the effect of brain structure and function and GABA levels in the visual-related cortex into consideration and improve the statistical efficiency of analyzing pattern glare levels and the influencing factors of visual memory. Fourth, the present study failed to provide direct evidence for improving visual memory since this was an association analysis in a case–control study. Longitudinal follow-up study is needed to verify the diagnosis of unipolar depression and to observe the changes in pattern glare and visual memory with the improvement of depressive symptoms. Finally, we did not take any interventions in the present study which is still preliminary. Measures are to be taken to alleviate pattern glare after further research on this issue, which is expected to make possible the direct observation of the effect of pattern glare on visual memory.

## Conclusions

Our study on the relationship between pattern glare and visual memory in patients with MDD showed that MDD patients had elevated levels of pattern glare and visual memory impairments. Moreover, visual memory was negatively associated with pattern glare in healthy controls. The underlying pathological mechanism of the association between high level of pattern glare and visual memory processing deserves further study.

## Supplementary Information

**Additional file 1: sTable 1.** Differences in pattern glare between MDD patients and HCs. **sTable 2.** Correlations between visual memory and pattern glare scores.

## Data Availability

The datasets generated and analyzed during the present study are not publicly available due to no permission from the ethics committee, but are available from the corresponding author on reasonable request.

## Abbreviations

MDD: Major depressive disorder
GABA: γ-Aminobutyric acid
HCs: Healthy controls
HAMD: Hamilton Depression Rating Scale
PRM: Pattern Recognition Memory
CANTAB: Cambridge Neuropsychological Test Automated Battery
BVMT-R: Brief Visual Memory Test-Revised
WAIS-RC: Wechsler Adult Intelligence Scale-revised China
