# Supplementary material for: The pattern glare and visual memory are disrupted in patients with major depressive disorder
Source: BMC Psychiatry. 2022 Aug 2;22:518. doi: 10.1186/s12888-022-04167-9 (PMC9344705; doi:10.1186/s12888-022-04167-9)
Supplement: Supplementary file 1 — Additional file 1: sTable 1. Differences in pattern glare between MDD patients and HCs. sTable 2. Correlations between visual memory and pattern glare scores. [file 12888_2022_4167_MOESM1_ESM.docx]

**sTable 1. Differences in pattern glare between MDD patients and HCs**

| **Items** | **MDD (N=62)** | **HCs (N=49)** | **t** | **Cohen’s** d | **p^*^** |
| --- | --- | --- | --- | --- | --- |
|  | **Mean (SD)** | **Mean (SD)** |  |  |  |
| **Mid-SF** | 4.26 (2.54) | 1.82 (1.45) | 6.01 | 1.15 | **< 0.001** |
| **High-SF** | 3.55 (2.83) | 2.14 (2.08) | 2.97 | 0.56 | **0.006** |
| **Mid-high difference** | 0.71 (1.88) | -0.29 (2.01) | 2.07 | 0.52 | **0.01** |

p^*^, corrected by FDR correction.

**sTable 2. Correlations between visual memory and pattern glare scores**

| **Items** | | **MDD (N=62)** | |  | **HCs (N=49)** | |  | **All subjects (N=111)** | |
| --- | --- | --- | --- | --- | --- | --- | --- | --- | --- |
|  |  | **Mid-SF** | **Mid-High difference** |  | **Mid-SF** | **Mid-High difference** |  | **Mid-SF** | **Mid-High difference** |
| **PRM-PCi** | **r** | 0.046 | 0.247 |  | -0.054 | 0.111 |  | -0.122 | 0.087 |
|  | **p** | 0.732 | 0.059 |  | 0.724 | 0.462 |  | 0.208 | 0.37 |
| **PRM-MCLd** | **r** | 0.158 | -0.039 |  | 0.146 | 0.061 |  | 0.246 | 0.054 |
|  | **p** | 0.232 | 0.772 |  | 0.334 | 0.685 |  | **0.01** | **0.578** |
| **BVMT-R2** | **r** | 0.222 | 0.116 |  | -0.071 | **-0.317** |  | -0.012 | -0.167 |
|  | **p** | 0.091 | 0.383 |  | 0.641 | **0.032** |  | 0.903 | 0.085 |
| **BVMT-R3** | **r** | 0.202 | 0.092 |  | -0.105 | -0.061 |  | -0.004 | -0.023 |
|  | **p** | 0.125 | 0.486 |  | 0.487 | 0.685 |  | 0.967 | 0.816 |
| **BVMT-Rt** | **r** | 0.186 | 0.099 |  | 0.013 | -0.158 |  | -0.028 | -0.097 |
|  | **p** | 0.159 | 0.455 |  | 0.93 | 0.295 |  | 0.775 | 0.316 |

Abbreviations: MDD, major depressive disorder; HCs, healthy controls; PRM, Pattern Recognition Memory; PRM-PCi, PRM percentage correct (immediate); PRM-MCLd, PRM mean correct latency (delayed); BVMT-R, Brief Visual Memory Test-Revised.
